# An Efficient Multi-Channel Electrotactile Parameter Configuration Method for Personalized Teleoperation

**DOI:** 10.3390/biomimetics11050310

**Published:** 2026-05-01

**Authors:** Kaicheng Zhang, Kairu Li, Peiyao Wang, Yixuan Sheng

**Affiliations:** 1 School of Electrical Engineering, Shenyang University of Technology, Shenyang 110870, China; dianqizkc@smail.sut.edu.cn (K.Z.); peiyao.wang@smail.sut.edu.cn (P.W.); 2 State Key Laboratory of Robotics and System, Harbin Institute of Technology Shenzhen, Shenzhen 150001, China; shengyixuan@hit.edu.cn

**Keywords:** teleoperation, human–robot interaction, electrotactile devices, finite element analysis

## Abstract

Electrotactile feedback is a compact approach for providing tactile cues in robotic teleoperation, but personalized calibration remains time-consuming because tactile perception varies across users. To address this problem, this study develops a subject-informed multi-layer finite element model of fingertip electric-field distribution coupled with a neural-response model and proposes a simulation-derived configuration-ranking method termed the Perceived Correctness Score (*PCS*). A gradient boosting regression model is then used to recommend among 36 candidate electrode diameter–spacing combinations. Validation was conducted using a custom-developed 3 × 2 multi-channel fingertip electrotactile stimulation system in a shape/area recognition task involving six healthy subjects. The predicted *PCS* showed a moderate positive correlation with the measured mean recognition accuracy across configurations (Pearson *r* = 0.48, *p* < 0.05). The model achieved Top-1 exact matching for three of six subjects and Top-5 coverage for five of six subjects. Compared with conventional exhaustive psychophysical calibration, the proposed method reduced the average configuration time from 122.7 min to 16.0 min, corresponding to an efficiency improvement of 87.0%. These results show that model-guided ranking can substantially reduce the burden of individualized electrotactile configuration.

## 1. Introduction

With the rapid advancement of robotics, teleoperation has been extensively deployed in hazardous, extreme, or otherwise inaccessible environments [1], encompassing high-precision tasks such as construction robot manipulation [2], remote Internet-of-Things collaboration [3], and biological sample microinjection [4]. Comparable requirements for precise perception and control also arise in micromanipulation tasks under complex interference conditions, where robust target localization is essential for reliable operation [5]. More broadly, recent studies on complex mechatronic systems have shown that coordinated tracking and constraint-aware control remain important for reliable operation in intelligent electromechanical platforms [6,7]. Similar considerations also arise in robotic manipulation systems, where robust or neural-network-based control strategies are often needed to maintain stable task performance under servo constraints and dynamic conditions [8,9]. Tactile feedback enables operators to perceive robotic interactions and adjust manipulation forces accordingly, playing a critical role in enhancing operational accuracy, controllability, and immersion [10,11]. Electrotactile stimulation has emerged as a key enabling technology for teleoperation owing to its compact form factor, low power consumption, and rapid response characteristics [12]. Compared with conventional mechanical tactile systems, electrotactile stimulation directly activates cutaneous nerve endings through low-intensity electrical currents, thereby providing feedback with superior spatial resolution and sensitivity [13]. As the primary site of tactile perception, the fingertip is of particular importance; the stability and precision of its tactile feedback directly govern the operator’s fine motor control capability [14,15].

Recent work in human–robot interaction has further emphasized that safe, human-centric operation increasingly depends on adaptive interaction planning. Digital-twin-assisted HRI frameworks have been proposed for safer collaborative motion planning [16]. Related studies on robust human interaction recognition and assistive systems further suggest that reliable human-centered interaction increasingly depends on multisensor perception and information fusion [17,18]. Recent robot-interaction studies also show that stable regulation of contact force remains fundamental when robots interact with soft or uncertain environments [19]. Neuroergonomics research further indicates that perceptual performance in fast-paced tasks depends not only on stimulus availability but also on how sensory information is integrated under cognitive load [20]. These trends jointly motivate fingertip tactile interfaces whose parameters can be individualized rapidly and in a physiologically grounded manner.

Due to the limited contact area available on the fingertip, electrotactile stimulation is prone to spatial current spread and inter-channel crosstalk, thereby increasing the difficulty of achieving reliable tactile localization. To improve spatial localization and discriminability, researchers have primarily focused on optimizing electrode geometry, spatial coding strategy, and interface layout. Garenfeld et al. [21] compared multiple fingertip electrode designs and demonstrated that electrode geometry and arrangement significantly affect tactile perception and localization performance. Boldt et al. [22] investigated the influence of temporal stimulus characteristics on two-point discrimination ability, providing empirical guidance for electrode spacing selection. Isaković et al. [23] analyzed the effects of reference-electrode size and position on fingertip electrotactile localization, confirming that appropriate size and position configurations can improve localization accuracy. Regarding multi-channel stimulation, Dosen et al. [24] proposed a multi-channel electrotactile scheme combining spatial and hybrid coding strategies, and subsequent work further showed that dual-parameter modulation can enhance localization performance [25]. Recent surveys also indicate that practical electrotactile interface quality depends not only on waveform selection but also on how electrode geometry, channel interaction, and wearable implementation are co-designed for the target application [12]. Collectively, these studies indicate that configuration parameters such as electrode size, spacing, and arrangement are decisive factors governing the spatial effectiveness of tactile feedback. However, the determination of optimal configuration parameters still largely relies on exhaustive one-by-one experimental comparison, making it difficult to achieve efficient and reproducible rapid configuration within a large candidate space.

Inter-individual variability is also a critical factor affecting electrotactile spatial performance. The perception threshold and subjective sensation elicited by an identical electrode configuration often differ substantially across individuals, with such variability being associated with factors including age, sex, and skin impedance [26,27], thereby undermining the generalizability of electrotactile feedback schemes. To address this issue, existing systems typically employ psychophysical procedures for individualized calibration, adjusting stimulation current amplitude, pulse width, and frequency parameters on a per-subject basis to match perceptual characteristics [28,29]. However, such calibration procedures are generally conducted under a predetermined electrode configuration and primarily operate at the stimulation-parameter level, making it difficult to incorporate individual physiological characteristics for pre-calibration optimization and recommendation of electrode geometric parameters such as diameter and spacing.

In the present study, existing electrotactile evaluation methods mainly rely on psychophysical testing, in which candidate electrode configurations are compared experimentally under a fixed hardware setup. For the 36 candidate diameter–spacing combinations considered here, this requires one-by-one testing of all configurations and is therefore time-consuming. In contrast, the present study proposes a subject-informed electrode configuration recommendation framework and introduces the Perceived Correctness Score (*PCS*) as a simulation-derived score for ranking candidate configurations before psychophysical verification. Using only basic subject descriptors, such as sex, age, height, and weight, the subject-informed FEM–neural-response framework generates configuration-level electric-field and neural-response features for all candidate geometries and uses them to rank the candidate configurations. Therefore, the main contribution of the proposed method is that it adds a subject-informed computational screening step before exhaustive psychophysical comparison, rather than relying only on post hoc experimental comparison. In this way, the proposed framework is intended to reduce the burden of exhaustive one-by-one psychophysical calibration and to support rapid personalized electrotactile configuration, rather than to replace traditional psychophysical evaluation altogether.

## 2. Materials and Methods

### 2.1. Personalized Electrode Configuration Recommendation Methodology

**Figure 1:**
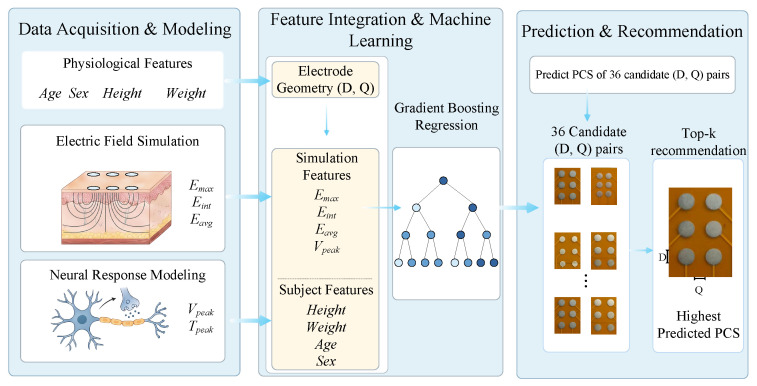
Overview of the subject-informed electrode configuration recommendation framework. Basic physiological descriptors are used to generate subject-specific electric-field and neural-response features for each candidate configuration, and a gradient boosting regression model is then used to predict *PCS* and rank the 36 candidate configurations.

This study proposes a personalized electrode configuration recommendation framework based on subject-informed finite element electric-field simulation, neural-response modeling, and machine-learning-based ranking. From an operational perspective, a new subject only needs to provide basic physiological descriptors, including sex, age, height, and weight. These descriptors are then used within the framework to generate individualized simulation features for each candidate configuration, after which a gradient boosting regression model predicts *PCS* scores and recommends the optimal electrode diameter–spacing combination. Thus, physiological descriptors are the external user inputs, whereas simulation-derived variables serve as internally generated intermediate features rather than additional experimentally measured inputs. The overall framework is shown in Figure 1.

#### 2.1.1. Model Input and Output Data

Electrode configuration parameters: The electrode diameter *D* (2.0–4.5 mm) and the edge-to-edge spacing *Q* between adjacent electrodes (0.5–3.0 mm) were selected as geometric design variables. Here, *Q* denotes the edge-to-edge spacing (i.e., the gap) between two adjacent electrodes rather than the center-to-center distance. Combinatorial enumeration at 0.5 mm increments yielded 36 candidate configurations, denoted as $D_{x}Q_{y}$, where *x* and *y* denote the electrode diameter and edge-to-edge spacing, respectively. These configurations span the geometric range relevant to fingertip electrotactile perception and constitute the candidate space for model prediction and recommendation.

External subject descriptors: The externally provided subject descriptors include sex, age, height, and weight. These easily measurable variables are used to parameterize the individualized fingertip model and condition the subsequent recommendation process.

Internal simulation-derived features and model output: For each candidate electrode configuration, the subject-informed FEM–neural-response pipeline automatically generates intermediate electric-field and neural-response descriptors, including $E_{\max}$, $E_{\mathrm{int}}$, $E_{\mathrm{avg}}$, $V_{\mathrm{peak}}$, and $T_{\mathrm{peak}}$. These variables are not additional measurements collected from the subject; rather, they are computationally derived internal features used to characterize each configuration under the subject’s physiological condition. The output of the framework is the predicted *PCS*-based ranking of the 36 candidate configurations, from which the highest-priority electrode geometry is recommended for subsequent verification.

#### 2.1.2. Electrode Parameter Configuration and Modeling

To achieve personalized recommendation, the electric field distribution and neural response on each individual’s skin were computed for each configuration to provide the physical basis for *PCS* evaluation. To this end, this subsection introduces the skin electrical parameter modeling method, employing the Cole-Cole model [30,31] to simulate skin conductivity. Considering the influence of individual physiological differences on skin electrical properties, the model assigns subject-specific electrical parameters to each skin layer based on the subject’s physiological information, thereby ensuring that the simulation results reflect the effects of inter-individual variability on the electrotactile stimulation response.

In the actual implementation, the multilayer fingertip geometry was represented using fixed layer thicknesses of 0.029, 0.089, 1.380, and 3.500 mm for the stratum corneum, viable epidermis, dermis, and subcutaneous tissue, respectively. These geometric thicknesses were kept constant across simulations to provide a controlled structural basis for exhaustive comparison among the 36 candidate electrode configurations. Inter-individual variability was not introduced by reconstructing subject-specific histological thicknesses for each layer; instead, it was introduced through subject-specific electrical parameter settings in this fixed multilayer geometry before neural-response simulation. This simplification was adopted because the aim of the present study was configuration ranking and rapid screening using a representative fingertip model, rather than exact subject-by-subject histological reconstruction. Similar representative-model simplification strategies have also been used in tactile mechanotransduction studies to link tissue mechanics, afferent response, and psychophysical behavior at the population-model level [32].

The subject’s physiological information was therefore used to assign layer-specific electrical properties, including conductivity and relative permittivity, in the coupled FEM–neural-response pipeline. These subject-specific electrical properties changed the FEM-computed extracellular potential distribution that drives the neural-response simulation. As a result, the simulated neural-response features varied across subjects and configurations and provided the neural basis for *PCS* construction and interpretation. The effect of this subject-specific modeling was evaluated through psychophysical results across subjects rather than through direct neural recordings.

The biophysical fingertip model and the coupled simulation pipeline were implemented in Sim4Life. The present study does not include direct validation against experimental neural recordings or external neural-level gold-standard benchmarks. Therefore, the current validation should be interpreted as behavioral support for the practical usefulness of the subject-informed *PCS*-based ranking, rather than as direct neural-level validation. Future work will include neural-level benchmarking and broader external validation.

Specifically, the Cole-Cole model was used to describe the frequency-dependent conductivity of the stratum corneum, epidermis, dermis, and subcutaneous tissue, as follows:

(1) $$\sigma_{c}\left(f\right)=\frac{\sigma_{\infty }}{1+{\left(j\omega \tau \right)}^{1-\alpha }}+\sigma_{0}$$

where $\sigma_{\infty }$ and $\sigma_{0}$ are the high- and low-frequency conductivities, *τ* is the relaxation time constant, and *α* is the relaxation distribution parameter.

Based on these subject-specific skin electrical parameters, a three-dimensional finite element model of fingertip electrotactile stimulation was constructed. The fingertip skin was represented by four layers, from outermost to innermost: stratum corneum, epidermis, dermis, and subcutaneous tissue. A 3 × 2 array of six circular electrodes was placed on the skin surface as independent stimulation channels, while A-β nerve fibers were embedded in the dermis to simulate stimulation-evoked neural responses.

**Figure 2:**
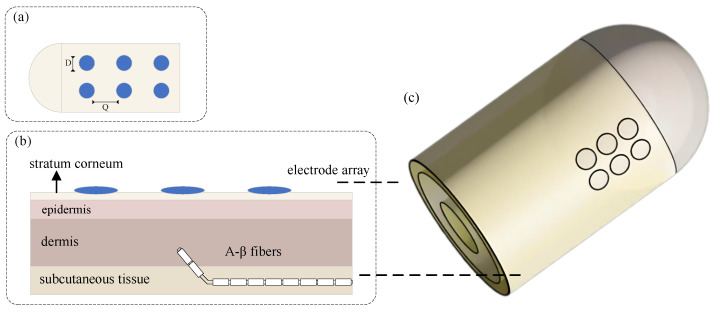
Three-dimensional finite element model of fingertip electrotactile stimulation. (**a**) Top view of the 3 × 2 circular electrode array, showing the electrode diameter *D* and edge-to-edge spacing *Q*. (**b**) Cross-sectional view of the multilayer fingertip model with four skin layers and embedded A-β nerve fibers. (**c**) Perspective view of the fingertip model and surface electrode array.

In the implemented neural model, each A-*β* fiber was assigned an axonal diameter of 9 μm. This value was chosen as a representative mid-range large myelinated cutaneous afferent diameter: large cutaneous A-*β* fibers in human skin are reported around 6–12 μm, and tactile-stimulation computational studies have also used 9 μm as a physiologically plausible A-*β* sensory fiber size [33,34]. Because the present work aims at configuration ranking rather than exact single-fiber electrophysiology, a single representative diameter was used for all subjects to avoid introducing an additional poorly identified free parameter. The detailed structure is shown in Figure 2.

(1) Electric field distribution. Based on the subject-specific skin electrical properties and electrode configuration parameters described above, the extracellular electric potential distribution within the fingertip tissue was computed using the finite element method (FEM) [35]. The governing equation is

(2) ∇·σ∇Φ=0

where *σ* denotes the electrical conductivity of the tissue and Φ denotes the extracellular electric potential. The electric field was obtained as the negative gradient of the electric potential, and three features of the electric field magnitude were extracted: the maximum value $E_{\max}$, the spatial integral $E_{\mathrm{int}}$, and the volume average $E_{\mathrm{avg}}$.

(2) Neural response. Previous studies have shown that combining neural fiber response analysis with computational modeling can improve the efficiency of electrical stimulation parameter optimization [36]. In this study, the SENN model [37] was used to compute the time-varying transmembrane voltage along the nerve fiber, as follows:

(3) $$\frac{dV_{n}}{dt}=\frac{1}{C_{m}}\left[G_{a}\left(V_{n-1}-2V_{n}+V_{n+1}\right)-G_{m}V_{n}\right]$$

where $V_{n}$ is the transmembrane voltage at the neural node, $C_{m}$ is the transmembrane capacitance, $G_{a}$ and $G_{m}$ are the internal and external conductances of the nerve fiber, respectively, and $V_{n-1}$ and $V_{n+1}$ are the transmembrane voltages at the adjacent nodes.

The extracellular potential $V_{e,n}\left(t\right)$ defined in Equation (5) provides the nodal values used in the driving term of Equation (3).

In the classical SENN model, the external driving potential is typically derived from the analytical solution for a point electrode in a homogeneous medium, which neglects the influence of the multilayer structure of the fingertip on current diffusion and may lead to discrepancies between the estimated and actual tissue potential distributions. To better represent current diffusion in layered tissue, the extracellular potential ϕ(x,t) was solved using the four-layer fingertip finite element model. The resulting potential was then mapped to each node of Ranvier along the A-β nerve fiber as the external input $V_{e,n}\left(t\right)$, thereby coupling the finite element electric field simulation with the neural response model. The electric field and extracellular potential are related by

(4) E(x,t)=−∇ϕ(x,t)

(5) $$V_{e,n}\left(t\right)=\phi \left(x_{n},t\right)=-\int_{C_{n}}E\left(l,t\right)\;dl$$

where $x_{n}$ is the position of the *n*-th node of Ranvier, and $C_{n}$ is the path from $x_{n}$ to the reference point. In the coupled implementation, the quantity $\left(V_{e,n-1}-2V_{e,n}+V_{e,n+1}\right)$ serves as the effective extracellular driving input to the neural-response calculation, and its magnitude influences whether an action potential is triggered.

In the present study, the neural response model is not used as a direct substitute for invasive neural recording. Instead, it serves as a biophysically informed computational step that converts the simulated electric-field input into neural-response features for *PCS* calculation and configuration ranking. Its practical usefulness is evaluated by comparing the PCS-based ranking with the measured psychophysical performance across the 36 electrode configurations in the six-subject shape/area recognition experiment.

#### 2.1.3. Electrode Configuration Performance Evaluation Method System

To transform the multidimensional simulation features into a quantitative score for evaluating electrode configurations, this study introduces a comprehensive evaluation method, termed the Perceived Correctness Score (*PCS*), based on the electric field distribution and neural response features obtained in Section 2.1.2.

The *PCS* integrates electric field and neural response features into a single score that reflects the expected spatial discrimination capability of each candidate configuration for a given subject. In constructing *PCS*, three aspects of configuration performance are considered: electric field focusing in the tissue, effective activation of afferent nerve fibers in the dermis, and the balance of the exported electric-field descriptors for the same configuration. These three aspects are combined in the following formulation:

(6) $$\mathrm{PCS}=w_{\mathrm{neuro}}\times u_{\mathrm{pattern}}\times \log\left(1+E_{\mathrm{focus}}\right)$$

The *PCS* evaluates the spatial discrimination potential of an electrode configuration through three components: electric field focusing ($E_{\mathrm{focus}}$), effective neural activation ($w_{\mathrm{neuro}}$), and descriptor balance ($u_{\mathrm{pattern}}$). The three components are combined multiplicatively, so that a low value in any one component lowers the overall *PCS*. A higher *PCS* indicates that the configuration is more likely to provide clear discrimination among stimulation channels.

Equations (6)–(10) constitute the authors’ proposed composite ranking method; only the underlying electric-field quantities and neural-response variables are inherited from the established FEM and SENN formulations cited above.

Unlike conventional electrotactile evaluation procedures that rely primarily on post hoc psychophysical thresholds or localization accuracy under a fixed hardware design, the proposed *PCS* is intended as a simulation-derived pre-screening metric. It combines electric-field focusing, neural activation sufficiency, and the balance of exported field descriptors into a single configuration-ranking score, thereby providing a physically informed prior for reducing the experimental search space before human validation.

$E_{\mathrm{focus}}$ represents the focusing degree of the electrode configuration, and is calculated as:

(7) $$E_{\mathrm{focus}}=\frac{E_{\max}}{E_{\mathrm{avg}}+\epsilon }$$

where $E_{\max}$ denotes the maximum value of the electric field, $E_{\mathrm{avg}}$ denotes the volume average of the electric field, and $\epsilon =10^{-9}$ is a small constant introduced to prevent division by zero.

$w_{\mathrm{neuro}}$ represents the neural response weight and is computed as:

(8) $$w_{\mathrm{neuro}}=\frac{1}{1+e^{-(V_{\mathrm{peak}}-V_{\mathrm{th}})\cdot k}}$$

where $V_{\mathrm{peak}}$ is the transmembrane voltage peak at the neural node under electrical stimulation, $V_{\mathrm{th}}$ is the activation threshold voltage, and *k* is the slope parameter of the sigmoid weighting function. Within the SENN-based framework adopted in this study [37], the activation threshold was set to $V_{\mathrm{th}}=-55$ mV and the slope parameter was set to *k* = 0.05 mV^−1^. Here, *k* determines the steepness of the sigmoid transition around the threshold, i.e., how sharply $w_{\mathrm{neuro}}$ changes as $V_{\mathrm{peak}}$ approaches and exceeds $V_{\mathrm{th}}$, rather than changing the threshold itself.

$u_{\mathrm{pattern}}$ describes the balance of the exported electric-field descriptors for the same configuration. In the present implementation, it is not defined from channel-by-channel values. Instead, it is built from three global quantities obtained from the same FEM post-processing workflow: the maximum electric field $E_{\max}$, the volume-weighted average electric field $E_{\mathrm{avg}}$, and the electric-field integral $E_{\mathrm{int}}$. When these quantities are more balanced, $u_{\mathrm{pattern}}$ becomes larger, which helps prevent the *PCS* from being dominated by only one field descriptor. It is calculated as

(9) $$u_{\mathrm{pattern}}=\frac{1}{1+C}$$

where *C* is a spread term defined from the three exported field descriptors. A smaller *C* means that the three descriptors are more balanced, whereas a larger *C* means that one descriptor is more dominant and the descriptor set is less balanced. Such imbalance may bias the composite score and therefore lead to a lower $u_{\mathrm{pattern}}$.

The spread term is calculated as:

(10) $$C=\frac{\sqrt{\frac{1}{n}\sum {\left(x_{i}-\mu \right)}^{2}}}{\mu }$$

where n=3, $x_{i}$ denotes the absolute magnitude of each exported field descriptor for the same configuration, i.e., $|E_{\max}|$, $|E_{\mathrm{avg}}|$, and $|E_{\mathrm{int}}|$, and *μ* is their mean. In this study, *C* is used only as an internal balancing term in *PCS*. Its purpose is not to define a separate physical quantity but to reduce the effect of configurations in which one descriptor becomes much larger than the others. Therefore, this term is used only inside the same scoring formula for relative comparison among candidate configurations.

#### 2.1.4. Personalized Configuration Recommendation Method Based on Gradient Boosting Regression

To achieve rapid configuration recommendation for new individuals, this study constructs a gradient boosting regression model based on the subject-informed simulation framework described in Section 2.1.2 and Section 2.1.3. A separate five-subject training group, denoted as T1–T5, was used to build the simulation-derived training dataset. Their physiological information is summarized in Table 1.

For each training subject, the individualized FEM–neural-response pipeline implemented in Sim4Life was run under all 36 candidate electrode configurations. For every subject–configuration pair, electric-field simulation data and neural-response simulation data were first generated. From these simulation outputs, configuration-level electric-field and neural-response features were extracted, and the corresponding *PCS* value was then computed according to Equations (6)–(10). Thus, one *PCS*-labeled sample was obtained for each subject–configuration pair, yielding 180 training samples in total. These training samples were used for model selection and regression-model construction and were fully independent of the six subjects (S1–S6) used in the subsequent psychophysical experiment.

From the operational perspective of new-user recommendation, only the subject’s basic physiological descriptors need to be provided externally. These descriptors parameterize a subject-informed fingertip model with fixed geometry and individualized electrical parameters and are then used to automatically generate intermediate simulation-derived features for each candidate configuration. At the computational level, the regression model uses both the externally provided physiological descriptors and the internally generated simulation features to predict and rank *PCS* values for the 36 candidate configurations. The output is the predicted *PCS* value ${\mathrm{PCS}}_{i}^{\mathrm{pred}}$ for each electrode configuration. The internally generated simulation features include the electric field maximum $E_{\max}$, electric field integral $E_{\mathrm{int}}$, and volume average $E_{\mathrm{avg}}$ computed via Equation (2), as well as the transmembrane voltage peak $V_{\mathrm{peak}}$ and peak time $T_{\mathrm{peak}}$ computed via Equations (3) and (5). The externally provided physiological descriptors include the subject’s *Height*, *Weight*, *Age*, and *sex*. The computational feature vector is constructed as follows:

$$x=\left[\begin{array}{c} E_{\max},E_{\mathrm{int}},E_{\mathrm{avg}},V_{\mathrm{peak}},T_{\mathrm{peak}},\\ \mathrm{Height},\mathrm{Weight},\mathrm{Age},\mathrm{sex} \end{array}\right]$$

For clarity, the regression model was trained to predict *PCS* computed from simulation-derived features rather than psychophysical recognition accuracy. The model inputs were the subject descriptors and simulation-derived features listed above, and the regression target was the corresponding *PCS* value computed for each subject–configuration pair. In the present study, model selection was performed on the five-subject training dataset, and the resulting model was then evaluated on an independent six-subject human group (S1–S6) to assess whether the predicted configuration ranking was consistent with measured behavioral performance. Therefore, the present validation includes independent testing on subjects outside the training set, although broader validation in larger and more diverse populations is still needed.

For model selection, five-fold cross-validation was employed to evaluate the generalization performance of linear regression, K-nearest neighbor regression, random forest regression, and gradient boosting regression, respectively. In the implementation, the candidate search covered KNN with k=1,3,5,7, random forest models with 100, 300, and 500 trees, and gradient boosting models with $n_{\mathrm{estimators}}=100$, learning rate = 0.1, and max depth = 3, or with $n_{\mathrm{estimators}}=200$, learning rate = 0.05, and max depth = 4. The final gradient boosting regressor was selected by the lowest five-fold cross-validated MAE, which is consistent with the expected nonlinear interactions among subject descriptors, electrode geometry, and simulation-derived features in a relatively small structured dataset. Let ${\mathrm{PCS}}_{i}^{\mathrm{calc}}$ denote the *PCS* value of the *i*-th sample computed via Equation (6), ${\mathrm{PCS}}_{i}^{\mathrm{pred}}$ denote the model-predicted value, and *N* denote the total number of training samples. The evaluation method adopts the mean absolute error:

(11) $$\mathrm{MAE}=\frac{1}{N}\sum_{i=1}^{N}\left|{\mathrm{PCS}}_{i}^{\mathrm{calc}}-{\mathrm{PCS}}_{i}^{\mathrm{pred}}\right|$$

**Figure 3:**
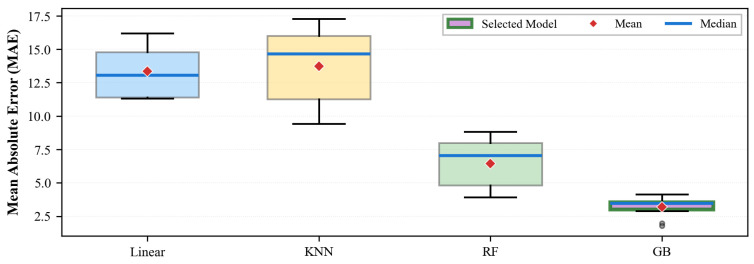
Comparison of regression model prediction performance. The figure presents the MAE of each model on the *PCS* prediction task; a lower value indicates higher prediction accuracy.

As shown in Figure 3, gradient boosting regression achieved the lowest MAE with the most stable error distribution and was therefore selected as the final model.

The model was trained using the least squares loss function, optimized by minimizing the squared error between the predicted and computed *PCS* values [38]:

(12) $$L=\frac{1}{N}\sum_{i=1}^{N}{\left({\mathrm{PCS}}_{i}^{\mathrm{calc}}-{\mathrm{PCS}}_{i}^{\mathrm{pred}}\right)}^{2}$$

To verify the effectiveness of the prediction model in practical applications, this study validates the trend consistency between the predicted *PCS* values and the experimentally measured recognition accuracy of subjects. Let ${\mathrm{PCS}}_{j}^{\mathrm{pred}}$ denote the predicted *PCS* value for the *j*-th electrode configuration (j=1,2,…,36), ${\mathrm{ACC}}_{j}$ denote the recognition accuracy measured through psychophysical experiments, and $\bar{\mathrm{PCS}}$ and $\bar{\mathrm{ACC}}$ denote the corresponding means. The Pearson correlation coefficient is employed to assess the trend consistency between the two:

(13) $$r=\frac{\sum_{j=1}^{36}\left({\mathrm{PCS}}_{j}^{\mathrm{pred}}-\bar{\mathrm{PCS}}\right)\left({\mathrm{ACC}}_{j}-\bar{\mathrm{ACC}}\right)}{\sqrt{\sum_{j=1}^{36}{\left({\mathrm{PCS}}_{j}^{\mathrm{pred}}-\bar{\mathrm{PCS}}\right)}^{2}\cdot \sum_{j=1}^{36}{\left({\mathrm{ACC}}_{j}-\bar{\mathrm{ACC}}\right)}^{2}}}$$

The *r* value reflects whether the predicted *PCS* and the experimentally measured recognition accuracy exhibit a consistent trend of variation across different electrode configurations.

Equations (11) and (12) are standard regression-error definitions used for model fitting and model selection, whereas Equation (13) is the standard Pearson correlation coefficient used here only for post hoc trend assessment between predicted configuration ranking and measured behavioral performance.

### 2.2. Six-Channel Electrotactile Stimulation System Design

#### 2.2.1. Hardware Design

**Figure 4:**
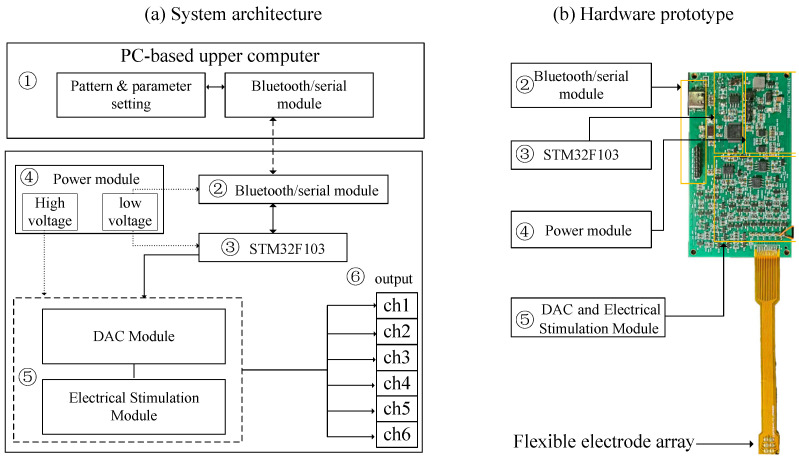
Hardware architecture and prototype of the six-channel fingertip electrotactile stimulation system. (**a**) System architecture, including the host computer, communication module, controller, power module, DAC and stimulation modules, and the flexible 3 × 2 electrode array. (**b**) Photograph of the PCB prototype with the main functional blocks labeled.

To experimentally validate the proposed personalized electrode configuration recommendation method, we developed a six-channel fingertip electrotactile stimulation system. The system integrates a communication module, a control module, a power supply module, and a stimulation driver module on a 92 mm × 50 mm printed circuit board, with the stimulation outputs connected to an external electrode array. The 3 × 2 electrode array is fabricated on a flexible printed circuit (FPC) substrate for conformal attachment to the fingertip. By varying the electrode diameter and edge-to-edge spacing, the effective coverage area of the array ranges from 0.315 cm^2^ to 2.34 cm^2^ within the fingertip contact area. The overall architecture is shown in Figure 4. The power module was designed to provide regulated supply paths for the controller, DAC, and stimulation driver, thereby reducing output fluctuation during channel switching and improving multichannel current-delivery stability.

During operation, the host computer transmits stimulation parameters and channel selection commands to the device via a serial link. The onboard controller then coordinates the power supply and stimulation driver to deliver six-channel biphasic current pulses through the selected electrodes. The device measures 92 mm × 50 mm and supports biphasic stimulation with a programmable amplitude of 0–10 mA (0.1 mA resolution), frequency of 1–100 Hz (1 Hz resolution), and pulse width of 10–500 μs (10 μs resolution).

#### 2.2.2. Software Design

**Figure 5:**
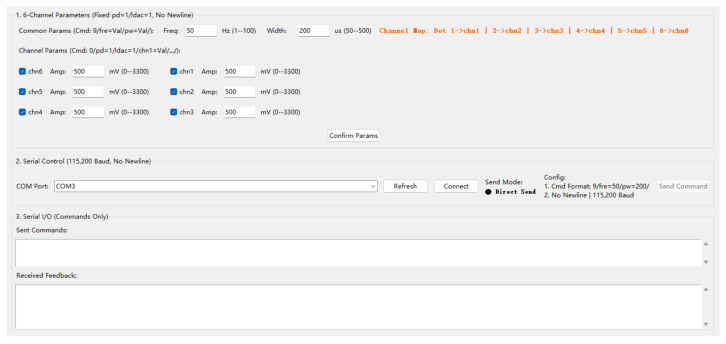
Graphical user interface of the host computer electrotactile stimulation control software. The interface enables unified configuration of stimulation frequency and pulse width, and independent amplitude adjustment for each channel.

The host computer software provides unified control of stimulation frequency and pulse width while allowing independent amplitude adjustment for each of the six channels. Users can activate any combination of channels through checkboxes and transmit the selected parameters to the stimulator in real time via the serial interface. As shown in Figure 5, the interface supports rapid adjustment of stimulation settings and flexible channel selection, thereby facilitating efficient execution of the psychophysical experiments.

#### 2.2.3. Psychophysical Experimental Protocol

The psychophysical evaluation followed a unified protocol designed to reduce between-subject baseline variability. Before formal testing, the index fingertip skin was cleaned, the FPC electrode array was attached to the dominant-hand index fingertip using conductive gel, and each subject completed a short familiarization session. Biphasic constant-current pulses were used in all formal tests, with frequency 1 Hz, single-phase pulse width 100 μs, stimulus duration 1 s, and inter-trial interval 3 s. Perception threshold was measured for each subject by an ascending method repeated three times, and the test amplitude was then set to 1.5 times the mean threshold. If contact quality changed noticeably during the session, the threshold was rechecked and the amplitude was updated accordingly. During testing, subjects wore blindfolds and headphones to minimize visual and auditory cues, and a 5 min rest was inserted after every 12 configurations to reduce fatigue and sensory adaptation. For method evaluation, all 36 candidate electrode configurations were experimentally tested for each subject. The model-generated Top-*k* recommendation was not used to terminate testing in this proof-of-concept study; instead, it was analyzed afterward to estimate how much the experimental search space could be reduced in a practical deployment scenario.

## 3. Results

This section evaluates the proposed personalized electrode configuration recommendation method through a shape/area recognition experiment. A 3 × 2 six-channel fingertip electrode array was tested under the 36 candidate configurations, i.e., different combinations of electrode diameter *D* and spacing *Q*, defined in Section 2.1.1. All participants were right-handed, healthy individuals without known sensory impairments. The study was approved by the Ethics Committee of Harbin Institute of Technology (Approval No.: HIT-2024077), and written informed consent was obtained from all participants.

By simulating fingertip contact with objects during teleoperation, this experiment evaluated whether the *PCS* model could recommend more suitable electrode diameter and spacing combinations while accounting for individual differences. Six right-handed healthy male subjects (S1–S6), aged 24–27 years and with no history of neurological disorders or finger skin injuries, were recruited. Their main physiological parameters are listed in Table 2. The experiment was conducted in a quiet, distraction-free environment.

**Figure 6:**
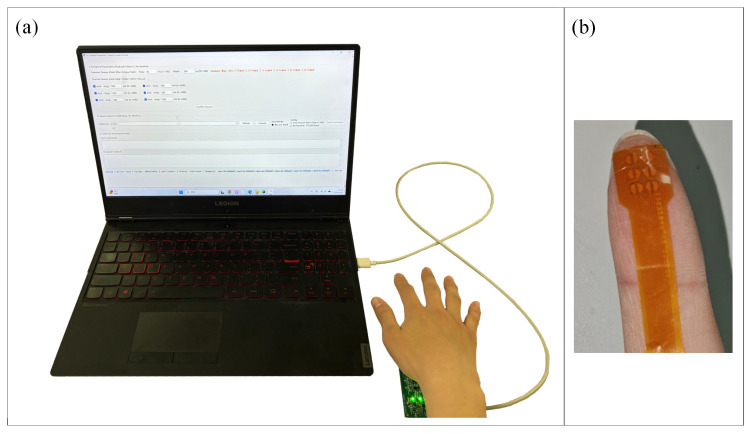
Experimental setup for the shape/area recognition experiment. (**a**) Overall view of the setup. (**b**) Close-up of the flexible 3 × 2 electrode array mounted on the index fingertip.

During the experiment, subjects kept their eyes closed and verbally reported spatial pattern judgments based solely on fingertip electrotactile feedback. An example of the setup is shown in Figure 6.

**Figure 7:**
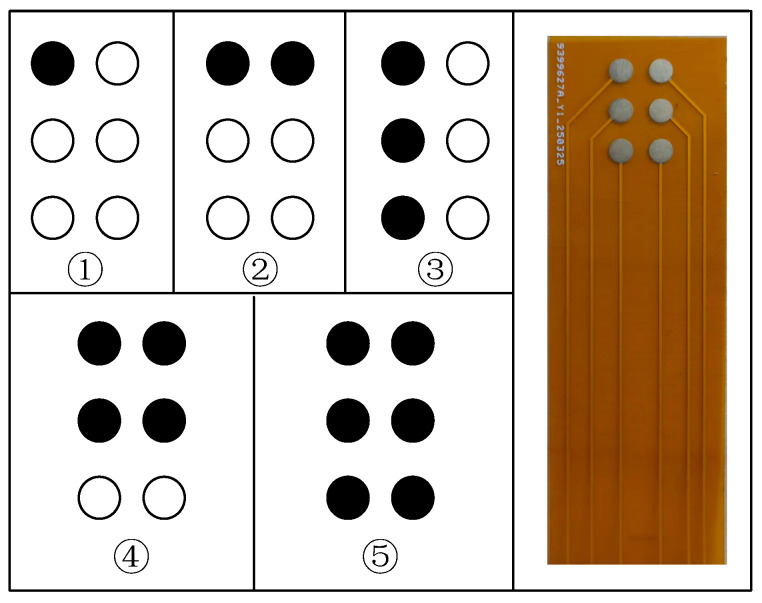
Schematic of the five shape/area recognition patterns used in the experiment: ① single point, ② horizontal line, ③ vertical line, ④ small area, and ⑤ large area. Black and white circles indicate active and inactive electrodes, respectively. The electrode layout is shown on the right.

The experiment employed five electrotactile spatial stimulation patterns: single point, horizontal line segment, vertical line segment, small area region, and large area region, simulating different spatial features perceived when the fingertip contacts objects, such as sharp points, edges, and flat surfaces. The electrode activation scheme for each pattern is illustrated in Figure 7. For each electrode configuration, subjects completed 15 recognition trials, i.e., the aforementioned patterns were randomly repeated three times, and subjects verbally reported the perceived pattern category. The testing order of the 36 electrode configurations was fully randomized; a 5-min rest was provided after every 12 configurations to mitigate fatigue effects.

Prior to the experiment, the *PCS* model computed and ranked *PCS* values for all 36 candidate electrode configurations to determine the priority set for verification, thereby compressing the experimental scope from full enumeration to a small number of high-priority candidates. Taking subject S1 as an example, the recommendation generation and verification results are presented in Table 3: the configuration D3.5Q1 was ranked as Top-1, while lower-ranked configurations such as D4Q3 appeared later in the recommendation list. After experimental verification of the configurations listed in the table, S1 achieved the highest recognition accuracy (80.00%) under the Top-1 configuration D3.5Q1, whereas lower-ranked configurations yielded relatively lower accuracy. These results demonstrate that *PCS* ranking can provide a reliable priority basis for configurations that are “more likely to achieve higher recognition performance,” thereby reducing the number of configurations requiring experimental verification and lowering the configuration cost.

**Figure 8:**
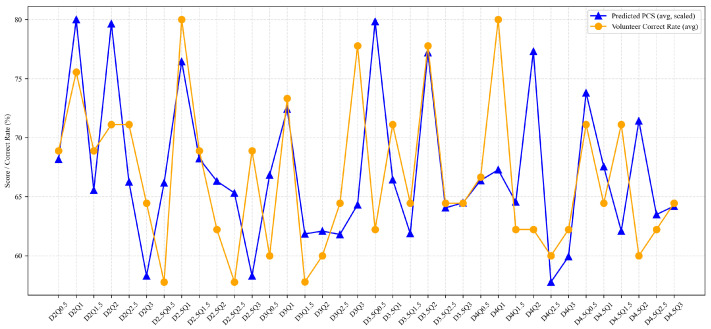
Comparison of predicted *PCS* and measured accuracy across 36 electrode configurations. The blue curve shows the predicted configuration-level *PCS*, and the orange curve shows the mean recognition accuracy across the six subjects.

To evaluate the ranking consistency of *PCS*, the predicted configuration-level *PCS* trend across the 36 electrode configurations was compared with the mean recognition accuracy across the six subjects, as shown in Figure 8. A moderate positive correlation was observed between the two (Pearson *r* = 0.48, *p* < 0.05), indicating that *PCS* provides preliminary evidence of configuration-level performance differences within the current group. Therefore, *PCS*-based ranking can be used to prioritize a small set of high-probability configurations for subsequent psychophysical verification, rather than replacing human validation altogether.

The recommendation results and experimentally measured optimal configurations for the six subjects are summarized in Table 4. Exact Top-1 matches were obtained for three subjects (S1, S3, and S4), corresponding to a Top-1 exact-match rate of 50.0%. For S2, the measured optimum D3.5Q2 appeared at Top-2; for S6, the measured optimum D4.5Q1 appeared at Top-5. For S5, the measured optimum D2Q2.5 appeared at Top-9 and therefore was not captured as the exact optimum within the Top-5 set. Nevertheless, the recommendation list still included the near-optimal configuration D3Q0.5, which achieved the second-highest measured performance for S5. Overall, the model achieved Top-5 coverage for 5 of 6 subjects (83.3%) in terms of exact-optimum matching, while the S5 case also suggests that the framework can still identify competitive near-optimal candidates even when the global optimum is not ranked within the Top-5 set.

The recommendation results summarized in Table 3 were obtained by applying the model selected on the five-subject training dataset to the independent six-subject test.

## 4. Discussion

(1) Validation of the effectiveness of the recommendation method.

The six-subject shape/area recognition experiment shows that the proposed *PCS*-based recommendation framework can effectively reduce the configuration search space. Exact Top-1 matching was achieved for 3 of 6 subjects (50.0%), while the experimentally optimal configuration fell within the Top-5 candidate set for 5 of 6 subjects (83.3%). Across the 36 candidate configurations, the predicted *PCS* showed a moderate positive correlation with the mean measured recognition accuracy (Pearson *r* = 0.48, *p* < 0.05). These results suggest that *PCS* can capture part of the configuration-level ranking trend within the present group and can therefore be used to prioritize high-probability candidates for rapid psychophysical verification. At the same time, the moderate magnitude of the correlation and the fact that the global optimum for subject S5 was not captured within the Top-5 set, although a near-optimal configuration was still included in the recommendation list, indicate that the current method should be interpreted as a ranking aid rather than as a full replacement for experimental calibration.

(2) Validation scope and group limitation.

Although the current validation included independent testing on a separate six-subject group, all test subjects were still young healthy males with relatively similar age and anthropometric ranges. Therefore, the present results still do not establish population-level generalizability. Future work should include larger and more diverse populations for broader external validation.

(3) Prediction deviation analysis and practical performance.

As shown in the trend plot (Figure 8), the *PCS* model’s predicted scores and the measured accuracy are generally consistent, although local deviations still exist for a small number of configurations. However, leveraging the two-stage configuration workflow of “Top-5 recommendation combined with limited experimental verification” adopted in this study, such local prediction deviations do not substantially affect the final application outcome. The specific analysis is as follows.

For configurations with relatively high predicted ranking but only moderate measured performance, the two-stage workflow remains useful because these candidates are examined within a small verification set rather than accepted automatically. In other words, prediction narrows the search space, whereas the final decision is still made by human-subject testing.

For the opposite case, i.e., when a configuration performs better experimentally than its predicted position would suggest, the current ranking may miss the global optimum for some users, as illustrated by subject S5. For S5, the measured optimum D2Q2.5 appeared at Top-9 and therefore was not captured as the exact optimum within the Top-5 set. Nevertheless, the recommendation list still included the near-optimal configuration D3Q0.5, which achieved the second-highest measured performance for S5. This suggests that the S5 deviation reflects a local ordering error among several competitive configurations rather than a complete failure to identify a practically useful candidate region.

Taken together, the practical value of the present framework lies in substantially reducing the experimental search space rather than guaranteeing exact ordering of all 36 candidates. Within that scope, the current results support the usefulness of combining predictive modeling with limited psychophysical verification for rapid individualized screening.

(4) Individual differences and the necessity of parameter adjustment.

The experimentally measured optimal configurations differed across the six subjects (D3.5Q1, D3.5Q2, D2Q2, D4Q2, D2Q2.5, and D4.5Q1), confirming that a single fixed diameter–spacing combination is unlikely to suit all users. These differences are consistent with the premise of the proposed framework: inter-individual variations in fingertip geometry, tissue properties, and perceived spatial spread affect the most favorable electrode geometry. By using subject descriptors to instantiate individualized simulation-derived features and then performing *PCS*-based ranking, the framework provides a subject-specific recommendation that can substantially reduce the number of configurations requiring manual testing.

(5) Relation to recent calibration and modeling studies.

Recent related models have shown that individual-specific or physiologically informed modeling improves electrotactile or transcutaneous-stimulation prediction and optimization. Yem et al. [27] concluded that individual skin impedance combined with machine learning improves fingertip current estimation. Hussain et al. [36] concluded that neural-response modeling can substantially improve optimization efficiency. RaviChandran et al. [39] showed that physiological fidelity is important for predicting neural excitation under transcutaneous stimulation. Consistent with these conclusions, our results also support the value of subject-informed modeling. More importantly, the present findings further show that subject-informed simulation outputs can be used not only for response prediction but also for configuration-level screening: the experimentally optimal configuration was included in the *PCS*-predicted Top-5 set for 5 of 6 subjects, and the average configuration time was reduced by 87.0%. Therefore, compared with similar recent models, the present work extends physiologically informed modeling from prediction or optimization to rapid multi-channel electrotactile configuration screening.

**Figure 9:**
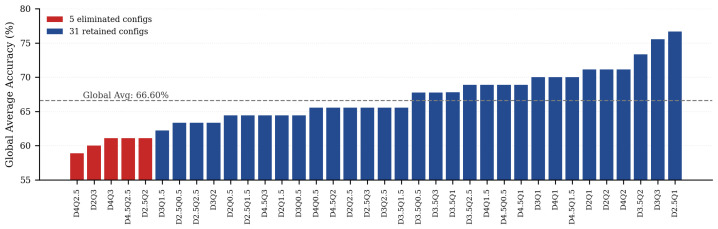
Global mean recognition accuracy across 36 electrode configurations in the six-subject shape/area recognition experiment. The gray dashed line indicates the overall mean accuracy, and excluded configurations are highlighted in red.

Reanalysis of the six-subject shape/area recognition dataset suggests a group-level empirical prior for reducing the configuration space. As shown in Figure 9, five configurations—D4Q2.5, D2Q3, D4Q3, D4.5Q2.5, and D2.5Q2—met the exclusion criterion of mean recognition accuracy < 61.5% and maximum subject accuracy < 80%. The overall mean recognition accuracy across all 36 configurations was 66.60%, and excluding these five low-performing configurations reduced the candidate set from 36 to 31 configurations.

The excluded configurations tended to cluster toward relatively large spacing settings, suggesting that excessively wide layouts may weaken stable spatial mapping within the limited fingertip area and reduce channel discriminability. Because each configuration was evaluated using a finite number of psychophysical trials (15 trials per subject), several configurations shared identical group-level mean accuracies; therefore, Figure 9 should be interpreted as supporting a threshold-based group-level pre-screening step rather than a fine-grained total ordering among all low-performing cases. Because this reduction was derived from a small homogeneous six-subject group, it should be interpreted as a practical pruning prior to the present study rather than as a universal elimination rule for all users or electrode layouts.

(6) Configuration efficiency.

The experimental time required for electrode configuration is primarily determined by the number of candidate configurations that need to be verified. The conventional exhaustive method requires completing identical recognition trials for all 36 configurations, with a total duration of approximately 120–125 min across the six subjects (mean: 122.7 min). In contrast, the *PCS* workflow first provides a small candidate set for targeted verification, thereby reducing the total duration to approximately 13–20 min (mean: 16.0 min) in the current group. This corresponds to individual savings ratios ranging from 83.5% to 89.2%, with an average improvement of 87.0% in configuration efficiency. These results demonstrate that the proposed workflow can substantially reduce the time burden of individualized configuration while preserving the same psychophysical task structure.

(7) Scalability and real-time implementation.

The present framework is mainly intended for rapid configuration screening rather than real-time closed-loop use. In the current implementation, subject-specific electric-field and neural-response features still need to be generated for the candidate configurations before *PCS* prediction, and this simulation stage remains computationally demanding. Therefore, although the regression-based ranking itself is lightweight, the overall recommendation process has not yet been optimized for real-time application. Extending the framework to denser arrays, broader geometric search spaces, or online closed-loop adaptation will further increase both computational cost and hardware/control complexity. Further work on scalability and practical real-time implementation will be included in our future research.

## 5. Conclusions

This study presents a personalized electrode configuration framework for multichannel fingertip electrotactile feedback in teleoperation-relevant scenarios. Using a custom 3 × 2 six-channel interface, we conducted a shape/area recognition experiment in six healthy subjects. Although the subjects kept their eyes closed and this task was not a full teleoperation experiment, the electrotactile patterns were delivered in random order and had to be identified immediately during stimulation. Therefore, the task still reflects real-time perceptual discrimination relevant to teleoperation. To overcome the inefficiency of conventional exhaustive psychophysical calibration, a subject-specific finite element model of fingertip electric-field distribution was coupled with a neural response model, and the Perceived Correctness Score (*PCS*) was introduced as a quantitative method for evaluating electrode configurations. A gradient boosting regression model was then employed to predict *PCS* values and rank 36 candidate diameter–spacing combinations, thereby prioritizing a small set of configurations for individualized verification. In the current six-subject group, the model achieved 50.0% Top-1 exact matching and 83.3% Top-5 coverage, while reducing the average configuration time from 122.7 min to 16.0 min (approximately 87.0% improvement). These results support the feasibility of using model-guided ranking to reduce configuration burden, although broader validation across more diverse participants is still needed. A teleoperation experiment incorporating a robotic hand and electrotactile feedback will be included in the next phase of our work.

Generalizability of the methodology and future extensions. Although the experimental validation in this study is currently limited to a six-channel electrode array uniformly distributed on the fingertip, the *PCS* prediction model and its accompanying “prediction–experimental verification” two-stage recommendation architecture establish a general methodology for tactile-stimulation parameter optimization. Different body sites (e.g., the forearm and torso) exhibit different nerve densities, spatial-discrimination limits, and inter-individual perceptual variability. The core logic proposed here—namely, first quantifying electrical diffusion and physiological constraints through computational modeling to reduce the physical parameter space, and then identifying a favorable individual configuration through a small number of human-in-the-loop psychophysical verification tests—is therefore transferable beyond a single hardware platform and a single test site. In future work, the same architecture can be extended to denser fingertip arrays or to individualized multi-channel electrode configuration tasks on other body parts such as the forearm. More broadly, this framework illustrates how physiology-informed computation can turn individualized electrotactile configuration from exhaustive search into guided screening.

## Figures and Tables

**Table 1:** Subject information for the simulation-based training dataset.

Subject	Sex	Age (Years)	Height (cm)	Weight (kg)	BMI
T1	Male	24	176	70	22.6
T2	Female	22	165	55	20.2
T3	Female	22	167	50	17.9
T4	Male	25	175	79	25.8
T5	Male	25	180	80	24.7

**Table 2:** Subject information for the shape/area recognition experiment.

Subject	Sex	Age (Years)	Height (cm)	Weight (kg)	BMI
S1	Male	25	177	72	23.0
S2	Male	26	178	81	25.6
S3	Male	24	173	60	20.0
S4	Male	25	175	80	26.1
S5	Male	27	174	64	21.1
S6	Male	25	178	82	25.9

**Table 3:** Example of personalized recommendation generation and verification for subject S1.

Model Input	Model Output (Recommended Scheme)		Experimental Verification	
Subject Features	Electrode Config.	Status	Accuracy	Evaluation
S1 Male, 25 yrs 177 cm, 72 kg	**D3.5Q1**	**Top-1**	**80.00%**	**Optimal**
	D3Q1	Top-5	73.33%	Effective
	D4.5Q0.5	Candidate	66.67%	Moderate
	D4Q3	Not recommended	53.33%	Poor

**Note:** Bold values indicate the optimal configuration and corresponding verification result for subject S1.

**Table 4:** Personalized recommendation results in the shape/area recognition experiment.

Subject	BMI	Predicted Config.	Best Measured	Accuracy	Hit
S1	23.0	D3.5Q1	D3.5Q1	80.00%	Top-1
S2	25.6	D4Q2	D3.5Q2	86.67%	Top-2
S3	20.0	D2Q2	D2Q2	93.33%	Top-1
S4	26.1	D4Q2	D4Q2	93.33%	Top-1
S5	21.1	D3Q1	D2Q2.5	86.67%	Top-9
S6	25.9	D4Q2	D4.5Q1	86.67%	Top-5

## Data Availability

The original data presented in this study can be downloaded from https://ieee-dataport.org/documents/fefns-tens (accessed on 28 April 2026).
